# A Patient Presenting with Cardiac Tamponade and the Challenges of Finding Its Cause: A Cardiac Angiosarcoma

**DOI:** 10.1155/2018/2084390

**Published:** 2018-02-28

**Authors:** Roshanak Habibi, Negar Faramarzi, Alvaro J. Altamirano, Shahriar Dadkhah

**Affiliations:** ^1^Department of Internal Medicine, Presence Saint Francis Hospital, 355 Ridge Ave., Evanston, IL 60202, USA; ^2^Department of Internal Medicine, Advocate Illinois Masonic Hospital, 836 W. Wellington Ave., Chicago, IL 60657, USA; ^3^Department of Cardiology, Presence Saint Francis Hospital, 355 Ridge Ave., Evanston, IL 60202, USA

## Abstract

Primary malignancies of the heart are so rare that most of the available data come from case reports or large single-center-based studies, with the overall incidence of 0.02% in the United States. Diagnosis in case of an isolated pericardial effusion as presentation is challenging, and determining that an angiosarcoma is even more challenging. Here, we presented a rare case of pericardial angiosarcoma which presented to us with tamponade. The patient eventually was diagnosed through pericardiectomy. A multimodality approach was attempted to treat the cancer. The clinical details of such a unique disease entity inspired us to present it as a case report.

## 1. Introduction

Pericardial angiosarcoma is a rare form of primary malignancy of the heart. Due to its insidious nature, most cases present with advanced disease with poor outcome. Diagnosis is challenging, demanding a panel of experts. There is no guideline for treatment. Hereby, we present a unique case of tamponade caused by primary angiosarcoma.

## 2. Case Description

**Figure 1:**
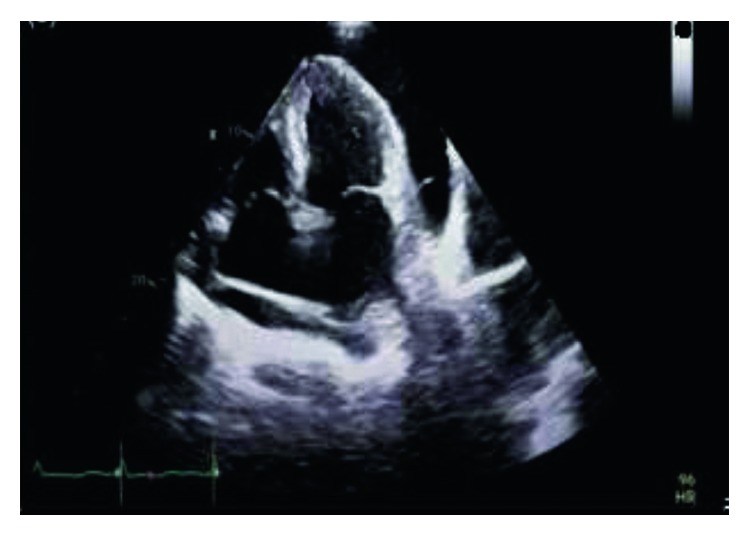
Two-dimensional TTE shows pericardial effusion in apical 4-chamber view.

**Figure 2:**
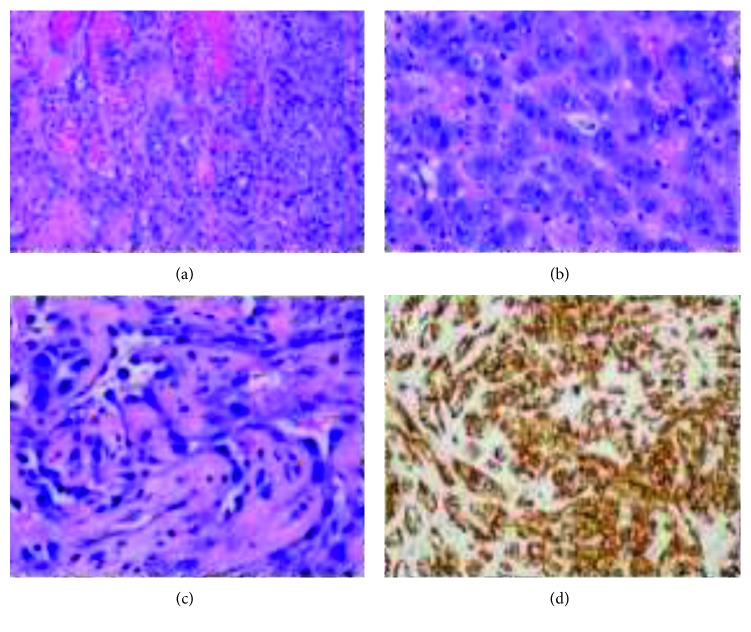
Hematoxylin and eosin stains of pericardium at 200x magnification (a) and at 400x magnification (b, c) demonstrate tumor cells with irregular nuclei arranged as interconnecting vascular space. Immunohistochemistry with positive staining for CD31 at 200x magnification confirms vascular differentiation (d).

On May 2016, a 59-year-old Bulgarian male referred from the office of cardiologist to our hospital complaining of worsening shortness of breath, weight gain of 6 pounds, leg swelling, and low blood pressures (BP) down to 80/60 mmHg for a few weeks. Medical history was significant for hypertension, coronary artery disease, diabetes, atrial fibrillation, polycystic kidney disease, and a recent tamponade diagnosed 4 months prior to this hospitalization. Dizziness was his initial complaint in March. A large serosanguinous pericardial effusion was drained at that time with inconclusive fluid analysis including a negative culture and cytology. His medications included amlodipine, lisinopril, and atorvastatin. He used to work as a truck driver. After 45 packs per year smoking history, he quit in 2015. Positive findings on exam were a BP of 85/60 mmHg, mild respiratory distress on room air, increased jugular vein pressure, muffled irregular heart sounds, and pitting edema of the shins. Echocardiography showed normal left ventricular ejection fraction of 59% and a moderate sized posterior pericardial effusion with borderline signs of tamponade. The echo also revealed a fibrinous material and stranding suggestive of adhesions in the collection (Figure 1). Attempt to drain the pericardial effusion with subxiphoid pericardial window was terminated due to fusion of pericardium to the anterior surface of right ventricle. Partial pericardiectomy was then attempted through median sternotomy with frozen section, which revealed a neoplastic process. Hence, a subtotal pericardiectomy was performed. There were adhesions of the pericardium to the right ventricular wall and diaphragmatic surface and nodularity on pericardium. Repeat echocardiogram showed no further effusion. Pericardium and epicardium tissue slides revealed predominantly solid sheets of high-grade epithelioid to spindle cells with focal areas of vasoformation. Tumor cells predominantly epithelioid show prominent atypia with abundant amophilic to eosinophilic cytoplasm, large vesicular nucleoli, and prominent nucleoli with atypical mitotic figures. On immunostaining, tumor cells were positive for CD31, CD34, WT1, vimentin, keratins AE1/AE3, and focal CK7 and negative for CK20, PSA, PSAP, AFP, TTF1, CDX2, BerEp4, calretinin, podoplanin D2, CD45, and S100 (Figures 2–2). These findings led to the diagnosis of epithelioid angiosarcoma of the pericardium. Chest computed tomography scan showed nodular opacifications within both lungs with concern for metastasis. His condition stabilized with Lasix and oxygen. He was then referred to the university hospital for a second opinion regarding his management. Cardiac MRI showed extensive vascular malignant mass in the pericardium encircling and infiltrating the right heart chambers. He received paclitaxel weekly for 6 doses with palliative intents. Later he developed malignant pleural effusions and intracranial metastatic lesions. He was admitted again in our hospital for weakness and encephalopathy. Patient preferred comfort care. Hence, he passed away 9 months after his initial presentation.

## 3. Conclusion

Primary pericardial neoplasms are rare entities with estimated prevalence of 0.001 to 0.007% based on mostly case series studies [1, 2]. Mesothelioma is the most common malignancy followed by different subtypes of sarcomas and lymphoma [1]. Angiosarcomas account for a third of sarcomas of the heart, and the majority arise in the right atrium. It can be seen at almost any age with peak incidence in middle-aged men. The majority are asymptomatic until they become large enough to cause symptoms. Presentation depends on area of involvement, such as emboli or obstruction in case of intracardiac lesion, arrhythmia with myocardial involvement, and pericardial effusion from pericardial raised tumor. Pericardial angiosarcoma may arise from the epicardial surface of the heart and penetrate the pericardial space. Signs of right-sided heart failure and pericardial effusion are common, which can lead to tamponade. In one study, pericardial effusion was common, but pericardial fluid cytology was negative in all patients who underwent pericardiocentesis [3]. By the time of diagnosis, most patients have metastases, most commonly to the lung [4]. Histologically, angiosarcomas consist of endothelial cells, spindle cells lining, and not well-defined anastomotic vascular spaces, causing sheet-like pericardial thickening. Malignant cells are positive for CD31, CD34, and factor VIII and form vascular channels confirming endothelial differentiation [5]. About 90% of patients die within the first year of diagnosis without resection [6]. Patients who receive multimodality treatment options (combination of surgery, radiation therapy, and chemotherapy) have improved survival compared to patients treated with only one modality. Even with all these strategies, longest survival remained less than 3 years [7]. Cardiac transplantation is not currently recommended due to poor survival benefit in few studies [8], except for very selected group of patients with subsequent immunotherapy [9]. Genome sequencing of the tumor in attempt to target gene therapy against vascular-specific receptor tyrosine kinases is currently being studied [2]. The optimal treatment strategy remains to be discovered. The diagnosis of heart angiosarcoma in our patient was challenging due to absence of a mass in different imaging of the heart, resulting in diagnostic pericardiectomy as only definite modality. Still, possibility of malignant tumor should be always considered in cases of recurrent serosanguinous pericardial effusions specially with a negative fluid cytology.

## 4. Summary

Primary malignancies of the heart are so rare that most of the available data come from case reports or large single-center-based studies, with the overall incidence of 0.02% in the United States. Diagnosis in case of an isolated pericardial effusion as presentation is challenging. Unique pathologic features include endothelial cells, spindle cells lining, and vascular spaces. Despite surgical approaches and different chemotherapeutic regimens, prognosis is poor with mean survival of up to 3 years. Different modalities such as heart transplantation, immunotherapy, and targeted gene therapy are recently being studied.
